# Genome-wide DNA methylation analysis of *Astragalus* and Danshen on the intervention of myofibroblast activation in idiopathic pulmonary fibrosis

**DOI:** 10.1186/s12890-023-02601-6

**Published:** 2023-09-04

**Authors:** Qingyin Liu, Xue Liu, Guoyu Wang, Fan Wu, Yuan Hou, Huaman Liu

**Affiliations:** 1 Shandong University of Traditional Chinese Medicine, No. 4655, Daxue Road, University Science Park, Changqing District, Jinan City, 250355 China; 2 Affiliated Hospital of Shandong University of Traditional Chinese Medicine, No. 16369, Jing Shi Road, Jinan City, 250013 China; 3 Capital Medical University, No. 10, Xizhang Road, Youanmenwai, Fengtai District, Beijing, 100069 China

**Keywords:** Idiopathic pulmonary fibrosis, Danshen, *Astragalus*, DNA methylation, Traditional chinese medicine

## Abstract

**Background:**

Idiopathic pulmonary fibrosis (IPF), a chronic progressive interstitial lung disease of unknown etiology, is characterized by continuous damage to alveolar epithelial cells, abnormal repair of alveolar tissue, and alveolar wall scar formation. Currently, the recommended treatment for IPF in Western medicine is relatively limited. In contrast, traditional Chinese medicine and compound prescriptions show advantages in the diagnosis and treatment of IPF, which can be attributed to their multi-channel and multi-target characteristics and minimal side-effects. The purpose of this study was to further corroborate the effectiveness and significance of the traditional Chinese medications *Astragalus* and Danshen in IPF treatment.

**Methods:**

We performed whole-genome methylation analysis on nine rat lung tissue samples to determine the epigenetic variation between IPF and non-fibrotic lungs using Gene Ontology and Kyoto Encyclopedia of Genes and Genomes pathway enrichment analyses and quantitative reverse transcription polymerase chain reactions.

**Results:**

We identified differentially methylated regions and 105 associated key functional genes in samples related to IPF and Chinese medicine treatment. Based on the methylation levels and gene expression profiles between the Chinese medicine intervention and pulmonary fibrosis model groups, we speculated that *Astragalus* and *Salvia miltiorrhiza* (traditionally known as Danshen) act on the *Isl1*, forkhead box O3, and Sonic hedgehog genes via regulation at transcriptional and epigenetic levels during IPF.

**Conclusions:**

These findings provide novel insights into the epigenetic regulation of IPF, indicate the effectiveness of *Astragalus* and Danshen in treating IPF, and suggest several promising therapeutic targets for preventing and treating IPF.

**Supplementary Information:**

The online version contains supplementary material available at 10.1186/s12890-023-02601-6.

## Background

Idiopathic pulmonary fibrosis (IPF) is a chronic, progressive interstitial lung disease of unknown etiology. Currently, the commonly accepted pathogenesis of IPF includes continuous damage to alveolar epithelial cells, abnormal repair of alveolar tissue, and alveolar wall scar formation [1]. The pathological hallmarks of IPF are disturbances in gaseous exchange in the lungs and restrictive ventilatory dysfunction. The median survival time after IPF diagnosis is two to three years [2]. In recent years, the role of alveolar epithelial cells in IPF has received increasing attention. Among them, when micro injuries occur repeatedly, dysfunctional alveolar type II epithelial cells (ATII) not only fail to maintain physiological lung regeneration but also promote abnormal epithelial interstitial crosstalk [3]. A computerized tomography (CT) imaging of IPF usually shows a typical usual interstitial pneumonia (UIP) pattern, characterized by irregular reticular opacity with mandatory honeycomb, which is associated with tractive bronchiectasis [4]. IPF occurs frequently among males, and its prevalence is directly proportional to age; additionally, a poor quality of life is commonly observed in patients. Recommended treatment measures for IPF in Western medicine guidelines are relatively limited and mainly include lung transplantation and anti-pulmonary fibrosis drugs, such as pirfenidone and nintedanib. These treatments have limitations, such as a high antibody titer ratio, shortage of organ donors, and severe adverse effects [5, 6]. Therefore, comprehensively investigating IPF pathogenesis and discovering new pathogenic genes and drug targets may be crucial for preventing and treating IPF. Genetic factors play a crucial role in the risk of IPF, and further research is needed to elucidate how these genetic factors guide clinical treatment decisions [7]. Research shows that IPF leads to abnormal changes in the messenger ribonucleic acid (RNA) (mRNA) and micro-RNA (miRNA) expression profiles [8, 9] as well as in a series of pathways including coagulation [10], apoptosis [11], oxidative stress [12], epithelial-mesenchymal transition [13–15], and other developmental pathways [14]. Increased local expression of coagulation factor X contributes to fibrosis response in human and mouse lung injury [10]. Autophagy alleviates the pathological progression of IPF by regulating apoptosis of fibroblasts and aging of alveolar epithelial cells [16]. Exogenous or endogenous reactive oxygen species (ROS) mediated oxidative stress directly damages alveolar epithelium [17]. Epithelial-to-mesenchymal transition (EMT) contributes to the early development of interstitial fibrosis through Paracrine signal transduction from alveolar epithelium to potential fibroblasts [18].

Recent studies have found that epigenetic phenomena widely occur in pulmonary fibrosis, which opens a new avenue for its prevention and treatment. The most prevalent type of epigenetic modification in eukaryotes is deoxyribonucleic acid (DNA) methylation, which regulates gene expression by changing the activity of non-coding elements [19], particularly 5-methylcytosine [20]. DNA methylation plays an important role in cell differentiation, tissue-specific gene expression, and chromosome inactivation [19, 21, 22]. A previous study elucidated that Thy-1, the fibroblastic foci in IPF, is expressed on normal lung fibroblasts but not on myofibroblasts; the downregulation of Thy-1 expression is attributed to promoter hypermethylation and histone modifications [23, 24]. With the development of sequencing technology, genome-wide differences in DNA methylation have been widely applied to study the differences between IPF and non-fibrotic lung [25, 26].

In recent years, traditional Chinese medicine (TCM) and compound prescriptions have shown advantages in IPF diagnosis and treatment, as they are multi-channeled, multi-targeted, and have few side effects. The combination of Chinese and Western medicines for IPF treatment has demonstrated good clinical effects [27]. Nevertheless, traditional Chinese medicine may also have some side effects and adverse reactions, but there is still a lack of high-quality and in-depth research at present. Previous clinical studies conducted by our research group have confirmed that qi and blood dysfunction occurred throughout IPF progression [28]. In the early stage of pulmonary fibrosis, inflammatory exudation is the main factor, with the intermingled qi, blood, and phlegm, obstruction of collaterals and blood stasis, and obstruction of arthralgia. In the middle and late stages of pulmonary fibrosis elevated fibrous tissue hyperplasia and extracellular matrix deposition are the main features. The normal structure of the alveoli is destroyed, and the lung function irreversibly disappears. At this stage, the lung lobes wither and the kidney are not used, the lung and kidney are deficient, the blood flow is stagnant, and the blood stasis is similar. We believe that although the focus of the pathogenesis of this disease varies in different periods, qi deficiency is the basic pathogenesis of this disease, and at the later stage, there is a tendency for heart and kidney yang deficiency, and blood stasis runs through the entire course of the disease. IPF can stimulate the occurrence of EMT and the thickening of extracellular matrix (ECM), thus making the gas blood barrier thicker. On the one hand, it can reduce the effective breathing area of the lung, and the places that could be used for gas exchange disappear. On the other hand, the remaining gas exchange tissue thickens, which is also not conducive to the process of lung exchange. Carbon monoxide is often used in clinical practice as a biomarker to measure diffusing capacity of the lungs for carbon monoxide (DLCO) to reflect lung ventilation function. Patients with diffusion dysfunction may experience varying degrees of symptoms such as wheezing, shortness of breath, and chest tightness due to difficulty in exchanging substances between air and blood because of problems with blood oxygenation. Qi deficiency and blood stasis are notable in IPF pathogenesis. Experiments on rats with bleomycin-induced pulmonary fibrosis showed that using *Astragalus* and Danshen effectively inhibited the proliferation of lung tissue fibers and matrix, intervened in myofibroblast activation, and delayed the progression of pulmonary fibrosis [29, 30].

The root of *Astragalus membranaceus*, a representative qi tonic medicine, has a distinct sweet taste and plays vital roles in regulating the lung and spleen meridians. *Salvia miltiorrhiza*, traditionally known as Danshen, has a bitter flavor profile and helps in mediating the heart and liver meridians, promoting blood circulation, and removing blood stasis. The combination of Danshen and *Astragalus* invigorates qi and activates blood circulation, which are important therapeutic targets in IPF. Data mining of TCM used in pulmonary fibrosis treatment has revealed that the most frequently used Chinese medicine for tonifying Qi and promoting blood circulation is *Astragalus membranaceus*, followed by Danshen [31]. This further corroborates the effectiveness and significance of *Astragalus* and Danshen in IPF treatment.

In this study, we performed whole-genome methylation analysis of nine samples of rat lung tissue to reveal the epigenetic variation between IPF and non-fibrotic lungs. We identified differentially methylated regions and associated key functional genes in samples related to IPF and Chinese medicine treatment. Our findings provide new possibilities for the prevention and therapy of IPF and highlight fresh insights into the epigenetic regulation of IPF.

## Methods

### Preparation of TCM

*Astragalus* (Huang qi formula) and Danshen formula particles were obtained from Beijing Tcmages Pharmaceutical Co., Ltd. (Beijing, China). Approximately 20 g of Huang qi formula particles are equivalent to 40 g of raw drug, whereas 10 g of Danshen formula particles are equivalent to 20 g of raw drug. The Chinese medicine-treated group was administered 40 g of raw Huang qi drug and 20 g of raw Danshen drug. The equivalent dose was converted to the equivalent dose ratio for humans and animals according to the body surface area [32]. The herbal formula particles were dissolved in 28 mL of warm water (30–40 ℃), resulting in an approximate concentration of 1.07 g/mL of the TCM solution.

### Animals and sample collection

Thirty distinct, pathogen-free seven-week-old Sprague–Dawley rats were grown in the laboratory. They were then randomly divided into three groups: the Chinese medicine intervention (TRE), pulmonary fibrosis model (MOD), and control (CON) groups. Under anesthesia, a single intratracheal instillation of 10.8 g/kg of each Huang qi formula and Danshen formula particles was administered to the TRE group. In contrast, under anesthesia, the MOD group received a single intratracheal instillation of 7 mg/kg bleomycin (Sigma, Rowe, NJ, USA), while the CON group received an equal volume of saline. Rats were euthanized after treatment for 28 d, and lung samples were harvested for further examination. The specimens were immediately frozen in liquid nitrogen and kept at -80 °C for future use during DNA extraction. All animal experiments were approved by the Institutional Animal Care and Use Committee of the Affiliated Hospital of Shandong University of Traditional Chinese Medicine (approval no. 81,804,034) and were performed in accordance with relevant guidelines and regulations. All methods are reported in accordance with Animal Research: Reporting of In Vivo Experiments (ARRIVE) guidelines (https://arriveguidelines.org) for the reporting of animal experiments.

### Masson staining

Lung specimens were fixed overnight with 4% Paraformaldehyde. The tissue sections were washed with Formaldehyde#Forms free water to remove excess Paraformaldehyde, dehydrate in a series of ethanol, and removed in xylene. Then they were embedded in paraffin and cut into 4 mm thick sections. To determine the degree of fibrosis, the sections were stained with Masson blue solution and examined under a microscope.

### Whole genome bisulfite sequencing (WGBS)

The samples were subjected to DNA extraction and subsequent WGBS analysis. Total genomic DNA was extracted using the QIAamp Fast DNA Tissue Kit (Qiagen, Dusseldorf, Germany), following the manufacturer’s instructions, and detected using a NanoDrop2000 ultraviolet-visible (UV-Vis) spectrophotometer (NanoDrop Technologies, Wilmington, DE, USA). Qualified DNA was fragmented by sonication and subjected to bisulfite conversion. Adapters were attached to single-stranded DNA fragments using an Accel-Next Generation Sequencing (NGS) Methylation Sequencing (Methyl-Seq) DNA Library Kit (Swift, Arbor City, MI, USA). The libraries were then sent to LC Sciences for pair-end 2 × 150 bp sequencing on an Illumina Hiseq 4000 platform (Hiseq 4000, Illumina, San Diego, CA, USA).

### Data quality control and analysis

The reads that included adapter contamination, undetected bases, and low-quality bases were eliminated using Cutadapt (version 4.0, Swedish National Bioinformatics Infrastructure) and Perl scripts (version 5.32.0, Perl Foundation, Portland, OR, USA) in-house [33]. Fast quality control (QC) was used to validate sequence quality. Clean data were mapped to the reference genome using Bisulfite Read Mapper and Methylation Caller (Bismark) [34], and the reads were further duplicated using Sequence Alignment/Map (SAM)tools [35].

The ratio of reads supporting certain phenomenon to the overall C-base reads was used to calculate the degree of DNA methylation at each cytosine nucleotide in the reference genome using custom Perl scripts and MethPipe. The R package “methylKit” was then applied to determine the differentially methylated regions (DMRs) with the following default parameters: 1,000 bp slide windows, 500 bp overlap, p value < 0.05, and |log2foldchange| ≥ 1.

### Gene Ontology (GO) and Kyoto Encyclopedia of genes and genomes (KEGG) pathway enrichment analyses

Genes situated within or nearest to the DMRs were identified and defined as differential methylation genes (DMGs). GO gene function and KEGG pathway enrichment analyses were applied to further filter DMGs and understand their functions. The top 20 GO terms and KEGG pathways were employed to perform functional enrichment analysis.

### Analysis of key functional DMGs

The intersection of the DMGs among the TRE, MOD, and CON groups was visualized using a Venn diagram. In addition, a heatmap was drawn to show the methylation levels of all DMGs using the R package. The intersection of the DMGs was then uploaded to the Search Tool for the Retrieval of Interacting Genes/Proteins (STRING) database to construct a protein-protein interaction (PPI) network. A medium confidence level of 0.4 was chosen as the interaction score. The DMGs in the PPI network were further filtered by degree values and regarded as key functional DMGs.

Methylation levels in the promoter regions of key functional DMGs were tested and compared with WGBS results. Furthermore, the expression profiles of key functional DMGs were detected using a quantitative reverse transcription polymerase chain reaction (qRT-PCR) to further understand the possible molecular regulatory mechanisms.

### qRT-PCR validation

Pursuant to the deoxidized RNAs (DE RNAs), competing endogenous RNAs (ceRNAs) were selected to substantiate the expression profiles in the CON and MOD groups using qRT-PCR. Here, validation was performed using the same RNA that was used for Illumina sequencing. A Roche LightCycler 480 (Roche, Forrentrasse, Switzerland) was used for qRT-PCR with SYBR Premix Ex Taq II (TaKaRa, Dalian, China). To standardize relative expression, the reference genes glyceraldehyde-3-phosphate dehydrogenase (*DAPDH*) and *U6* were used. Supplementary Table S1 lists the primers used in this study. Relative gene expression levels were analyzed using the 2^–ΔΔCt^ method. Statistical analyses were performed using Statistical Product and Service Solutions (SPSS) (version 20.0, International Business Machines Corporation [IBM], Armonk, NY, USA). Differences were considered statistically significant at p < 0.05.

## Results

### Validation of the animal model of pulmonary fibrosis

**Fig. 1:**
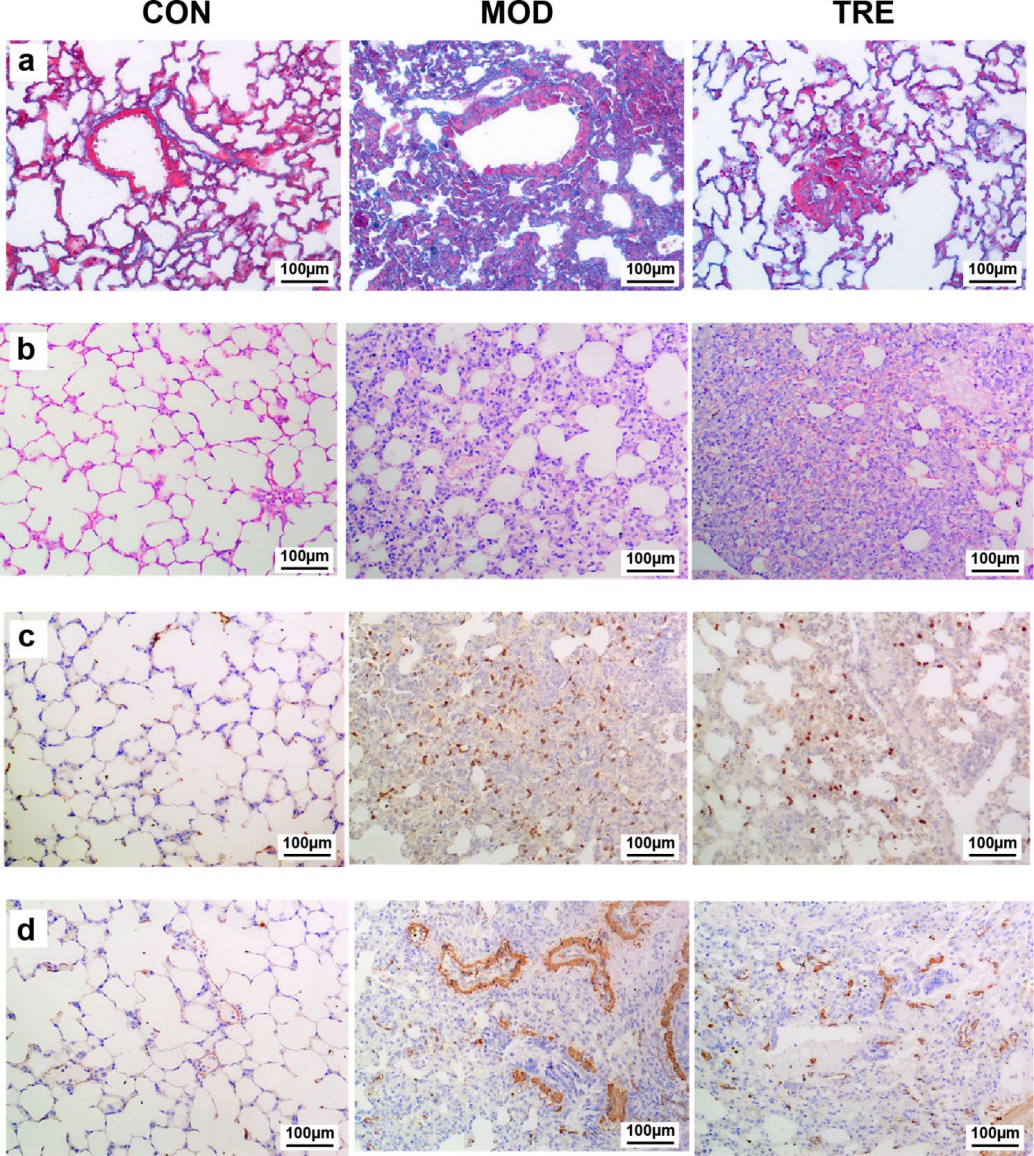
Validation of the pulmonary fibrosis animal model. (**a**) Sprague–Dawley (SD) rat pulmonary Masson sections. (**b**) SD rat lung tissue slices stained with hematoxylin and eosin (HE). (**c**) The immunohistochemistry (IHC) results for collagen I in SD rat pulmonary tissue sections. (**d**) The SD rat pulmonary IHC sections for α-smooth muscle actin (α-SMA)

Masson staining was conducted to successfully establish the pulmonary fibrosis rat model. The alveolar structures of the CON group lung specimens were intact and consecutive, with no conspicuous anomalies (Fig. 1). The alveolar septum was thinner and had fewer collagen fibers than did the other group specimens. In contrast, the MOD and TRE groups had numerous lung fibrous nodules (blue region) in the lung interstitium. The collagen fiber formation and collagen deposition areas were lower in the TRE than in the MOD group. The existence of the pulmonary fibrosis rat model was proven by all the aforementioned characteristics.

### Sequencing data quality control and mapping analysis

After quality control, valid data were obtained from nine samples. The Q20% range was between 94.64% and 95.75%, and the Q30% was between 87.81% and 89.79%. The guanine + cytosine (GC) content ranged between 21.28% and 22.95%. The conversion efficiency after the bisulfite treatment was greater than 99%. After mapping these data to the reference genome, the mapping data ranged between 52.23% and 66.59%. All these outcomes indicated that our data were of a high standard and useful for future research. The detailed data are shown in Table 1 and 2.

**Table 1:** Sequencing data quality control results

Sample	Raw Data		Valid Data		Valid%	Q20%	Q30%	GC%
	Read	Base	Read	Base				
CON_1	572,571,314	85.89G	570,813,220	84.71G	99.69	95.41	89.17	21.68
CON_2	827,093,422	124.06G	824,779,802	122.38G	99.72	95.57	89.49	21.68
CON_3	878,124,394	131.72G	876,145,056	130.02G	99.77	95.75	89.79	21.28
MOD_1	636,370,286	95.46G	634,473,170	92.53G	99.70	95.33	89.31	21.58
MOD_2	628,000,372	94.20G	626,334,352	91.00G	99.73	95.52	89.71	21.90
MOD_3	742,869,824	111.43G	740,839,860	107.48G	99.73	95.39	89.48	21.56
TRE_1	568,018,468	85.20G	567,623,822	82.11G	99.93	94.85	88.05	22.90
TRE_2	545,565,836	81.83G	545,181,794	78.58G	99.93	94.87	88.10	22.83
TRE_3	542,790,046	81.42G	542,271,926	78.17G	99.90	94.64	87.81	22.95

CON, control group; GC, GC base pairs; MOD, pulmonary fibrosis model group; TRE, Chinese medicine intervention group

**Table 2:** Sequencing data mapping to the reference genome

Sample	Total reads	Mapped reads	Mapping rate (%)	Duplication rate (%)	BS conversion rate (%)
CON_1	570,813,220	368,374,458	64.54	32.74	99.41
CON_2	824,779,802	540,075,344	65.48	28.76	99.38
CON_3	876,145,056	583,385,340	66.59	28.96	99.35
MOD_1	634,473,170	333,257,100	52.53	39.08	99.46
MOD_2	626,334,352	335,623,340	53.59	37.98	99.45
MOD_3	740,839,860	386,923,306	52.23	37.53	99.45
TRE_1	567,623,822	358,598,822	63.18	19.80	99.55
TRE_2	545,181,794	342,567,646	62.84	18.96	99.55
TRE_3	542,271,926	326,766,918	60.26	18.00	99.54

BS, bisulfite sequencing; CON, control group; MOD, pulmonary fibrosis model group; TRE, Chinese medicine intervention group

### DNA methylation patterns

As shown in Table 3, the methylation models of C-bases (CG, CHG, and CHH, where H is A, T, or C) were obtained and compared to the methylation contexts and percentages of the nine samples. The average methylation level of the C-base across the genome was between 3.47% and 3.75%. Most of these DNA methylation patterns were CG, and the percentage range of the nine samples was between 74.16% and 76.88%. In addition, the average methylation levels of CHG and CHH were similar: 0.27–0.53% and 0.27–0.5%, respectively. The results indicated that the sequencing depth in our study met these requirements.

**Table 3:** C-base methylation states

Sample	mCpG%	mCHG%	mCHH%	mC%
CON_1	0.7658	0.0051	0.0047	0.0363
CON_2	0.7688	0.0052	0.0049	0.0371
CON_3	0.7728	0.0053	0.0050	0.0369
MOD_1	0.7744	0.0050	0.0047	0.0360
MOD_2	0.7743	0.0050	0.0047	0.0375
MOD_3	0.7743	0.0050	0.0048	0.0368
TRE_1	0.7463	0.0031	0.0030	0.0352
TRE_2	0.7416	0.0031	0.0029	0.0347
TRE_3	0.7704	0.0029	0.0027	0.0352

CON, control group; m%, mean methylation levels; MOD, pulmonary fibrosis model group; TRE, Chinese medicine intervention group

### DNA methylation levels and distributions

**Fig. 2:**
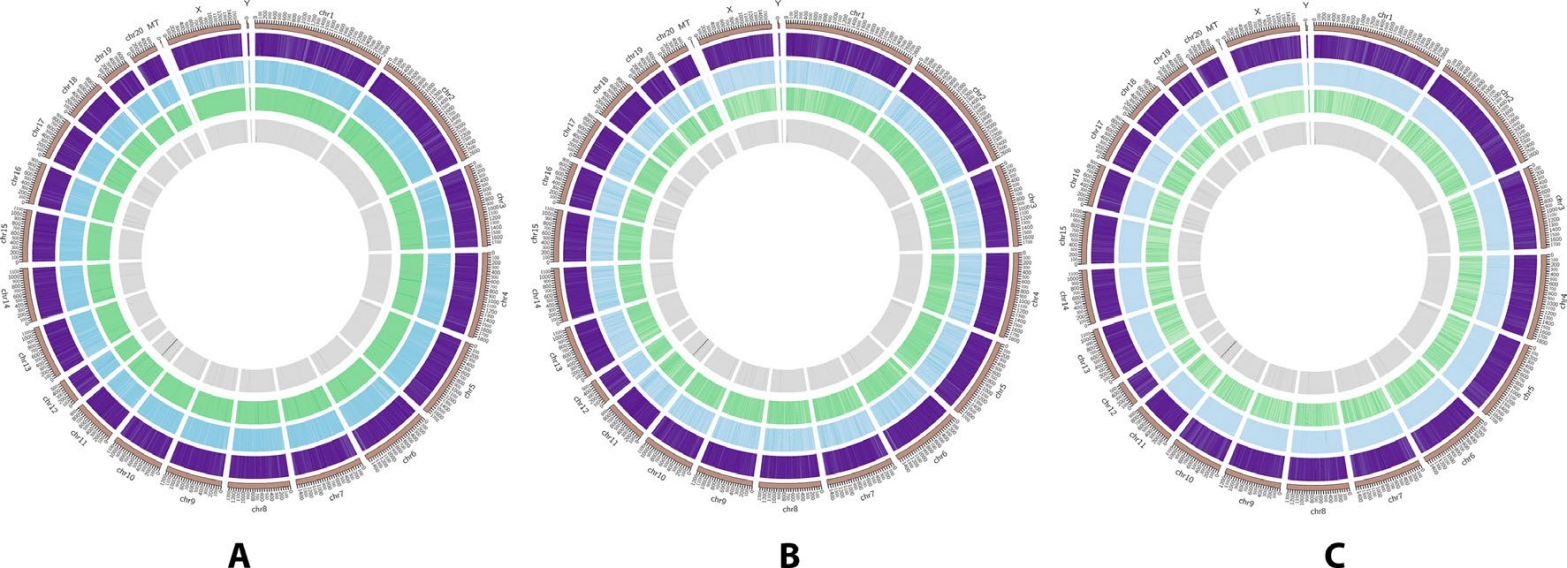
Methylation map at the chromosomal level

The methylation status of every chromosome was detected by sliding a 10 kb window and constructing a genome-wide methylation map at the chromosomal level to describe the distribution of methylated C-bases. As shown in Fig. 2, the methylation levels of base C differed among different chromosomes, with comparatively high levels in the CG patterns. Moreover, the number of genes involved was relatively large.

**Fig. 3:**
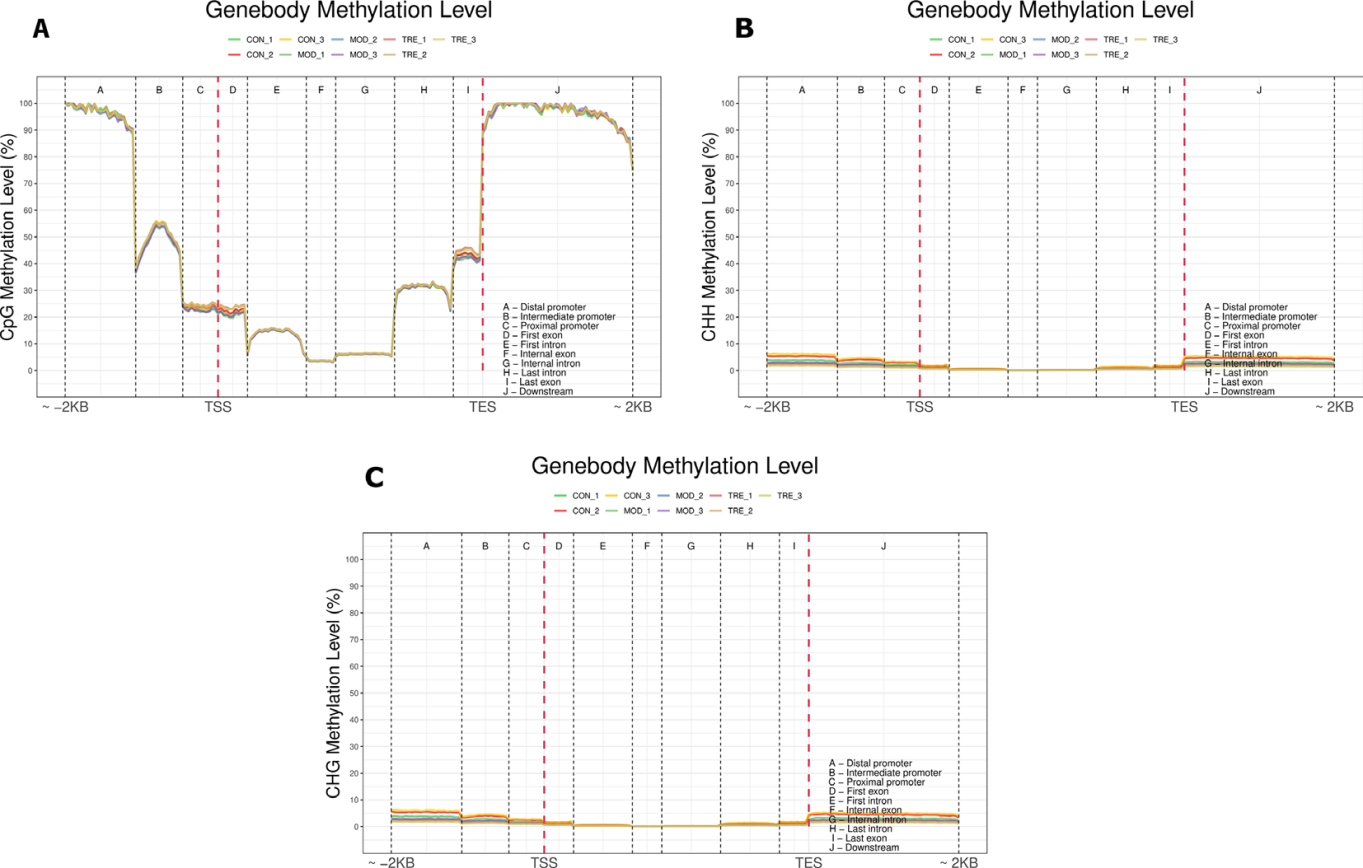
Methylation levels of different gene functional elements

The C-base methylation levels in different contexts were analyzed in different functional elements. The multiple cloning sites were divided into remote, middle, proximal, first, internal, last, and downstream regions. Regarding CG, the methylation levels of all three sample groups were high in the promoter region and low in the intron and exon regions. Methylation levels of all sample groups were low for all functional elements in the CHG and CHH contexts (Fig. 3).

**Fig. 4:**
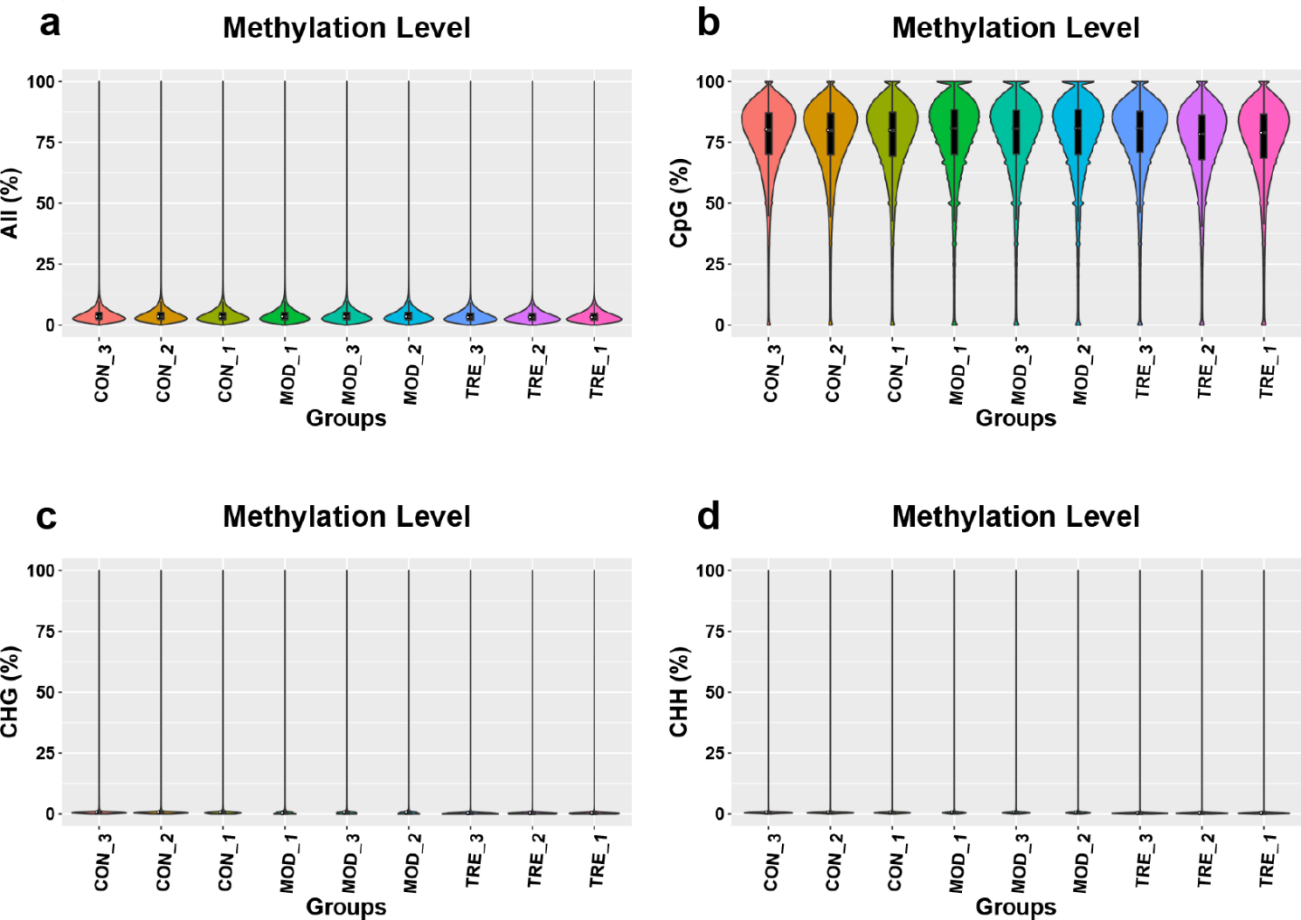
Methylation level distribution of samples

A violin plot was used to determine the C-base methylation level distribution of each group (Fig. 4). The methylation level distribution of the nine samples in each context (CG, CHH, and CHG) was similar. According to the cross-sectional area, the methylation level in the CG context was speculated to be higher than that in the CHH and CHG contexts.

### Analysis of DMRs

**Fig. 5:**
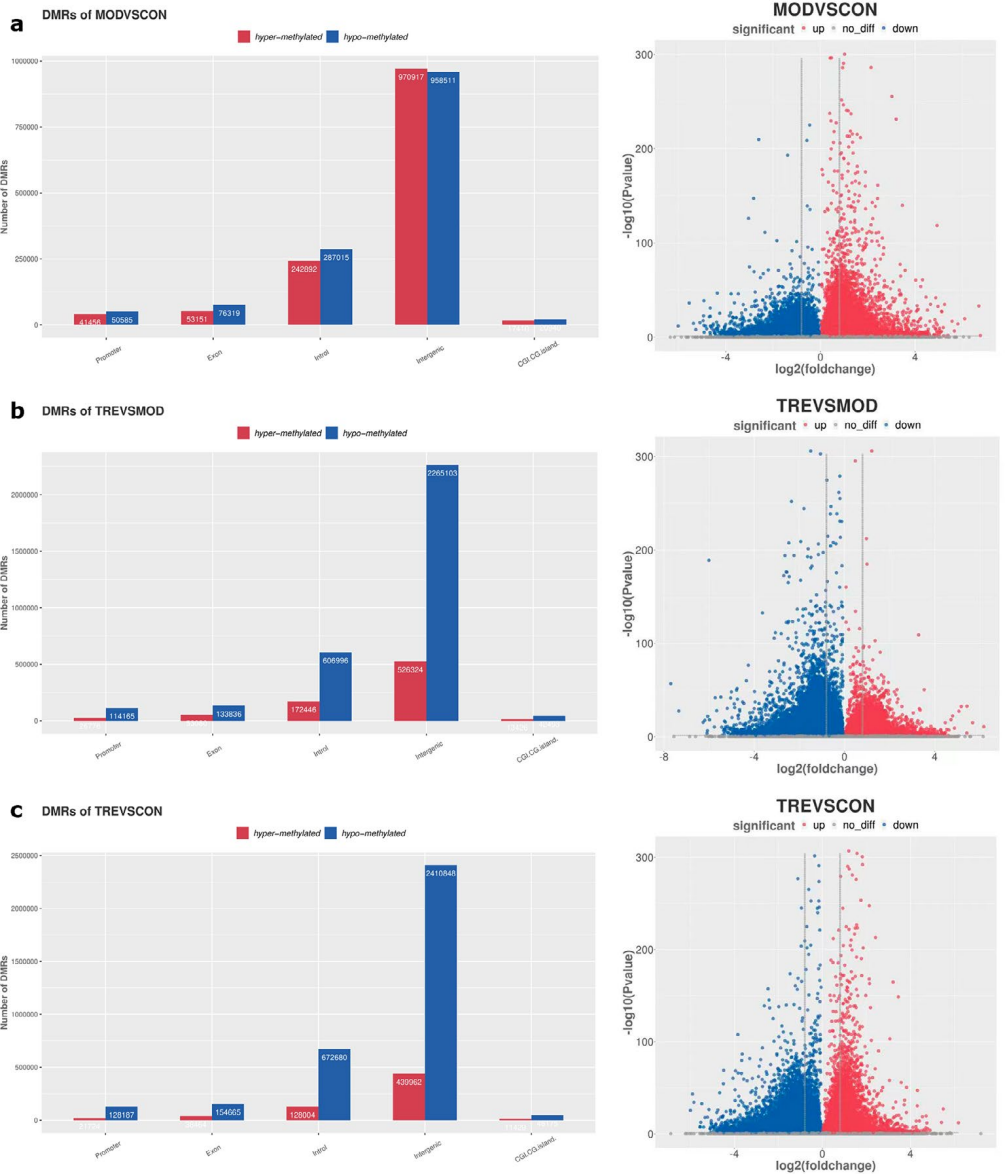
Differentially methylated regions between diverse groups

DMRs in the promoter region were detected using pairwise comparisons among the three groups. The results revealed 41,456 hypermethylated and 5,055 hypomethylated DMRs in the CON and MOD groups, respectively (Fig. 5a). Furthermore, 26,766 DMRs were hypermethylated and 114,155 were hypomethylated between the MOD and TRE groups (Fig. 5b). Moreover, 21,724 DMRs were hypermethylated and 128,187 were hypomethylated between the CON and TRE groups (Fig. 5c).

**Fig. 6:**
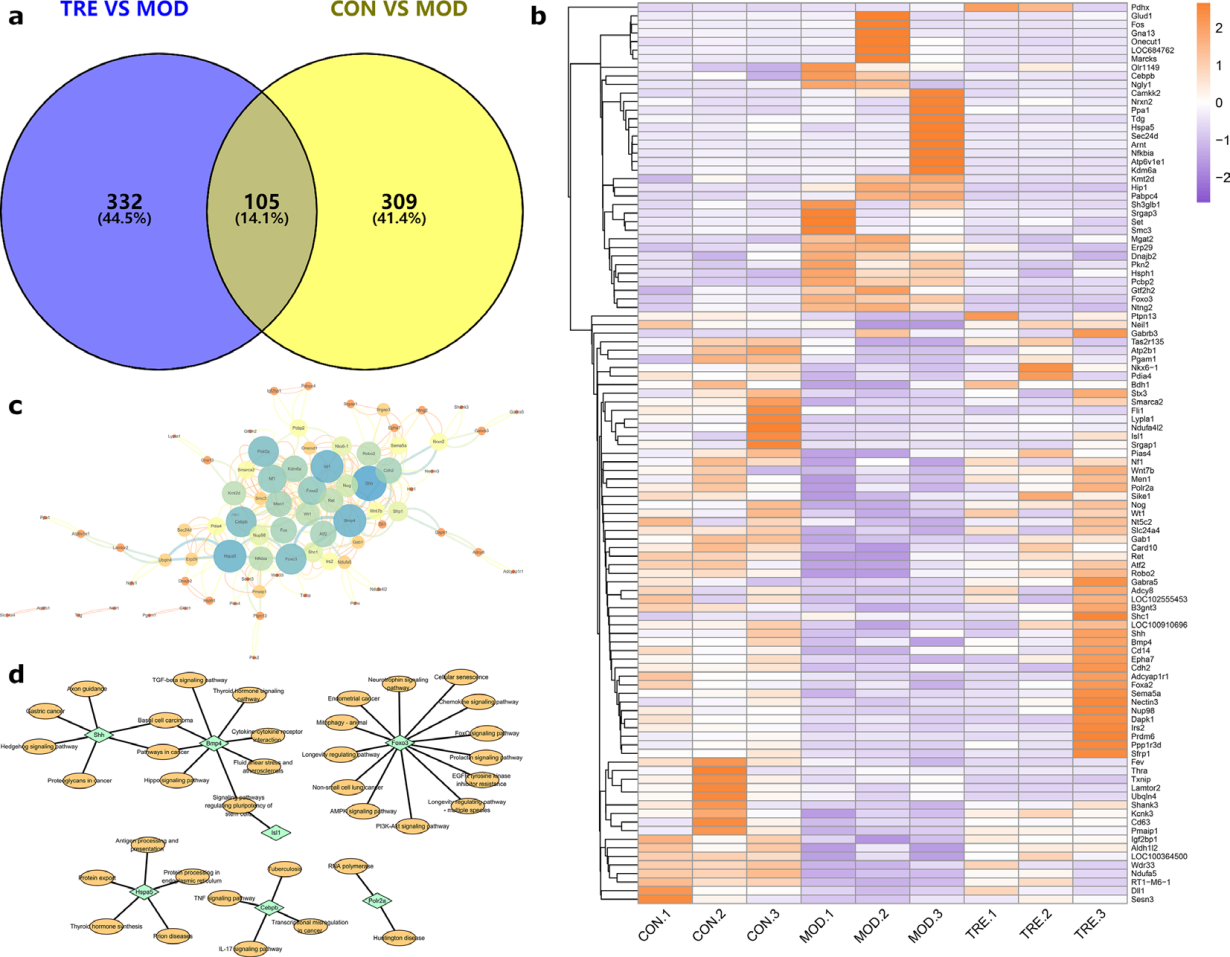
Differential methylation gene (DMG) screening and enrichment analysis. (**a**) Venn diagram of shared key DMGs. (**b**) Heatmap of key functional DMGs. (**c**) Protein-protein interaction network of DMGs. (**d**) Kyoto Encyclopedia of Genes and Genomes enrichment analysis of DMGs

Genes situated within or close to the DMRs of the intergenic area were delineated as DMGs. Venn diagrams were used to show the key DMGs shared by comparing the TRE vs. MOD and CON vs. MOD groups, following which we identified 105 DMGs (Fig. 6a). The heatmap in Fig. 6b shows the methylation levels of key DMGs. We found that in the TRE group, *Shc1*, gamma-aminobutyric acid type A receptor subunit 5 (*Gabra5*), *Nectin3*, insulin receptor substrate 2 (*Irs2*), protein phosphatase 1 regulatory subunit 3D (*Ppp1r3d*), *Shh*, bone morphogenetic protein 4 (*Bmp4*), cluster of differentiation 14 (*Cd14*), EPH receptor A7 (*Epha7*), cadherin 2 (*Cdh2*), adenylate cyclase activating polypeptide receptor type 1 (*Adcyap1r1*), forkhead box A2 (*Foxa2*), Semaphorin 5 A (*Sema5a*), nucleoporin 98 precursor *(Nup98), death-associated protein kinase 1 (Dapk1), and Prdm6 were hypermethylated*, whereas DnaJ homolog subfamily B member 2 (*Dnajb2*), endoplasmic reticulum protein 29 (*Erp29*), *FOXO3*, and huntingtin interacting protein 1 (*Hip1*) were hypomethylated.

A PPI network of 105 key functional DMGs was constructed using the STRING database. Cytoscape was used to process and visualize the data. After processing, 78 DMGs were applied to form a PPI network (Fig. 6c), and seven DMGs (RNA polymerase II subunit A [*Polr2a*], *Isl1, FOXO3*, CCAAT enhancer binding protein beta [*Cebpb*], heat shock protein family A (Hsp70) member 5 [*Hspa5*], *Shh*, and *BMP4*) were filtered by degree value for KEGG pathway enrichment analysis. The results showed that the tumor necrosis factor (TNF), autophagy, apoptosis, cyclic adenosine monophosphate (cAMP), Hippo, phosphatidylinositol-3-kinase (PI3K)-Akt, interleukin 17 (IL-17), transforming growth factor beta (TGF-β), and FOXO signaling pathways were involved (Fig. 6d).

**Fig. 7:**
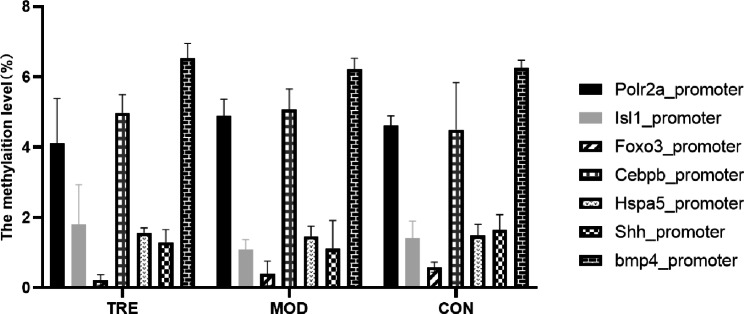
Methylation levels of key DMGss in the promoter region

**Fig. 8:**
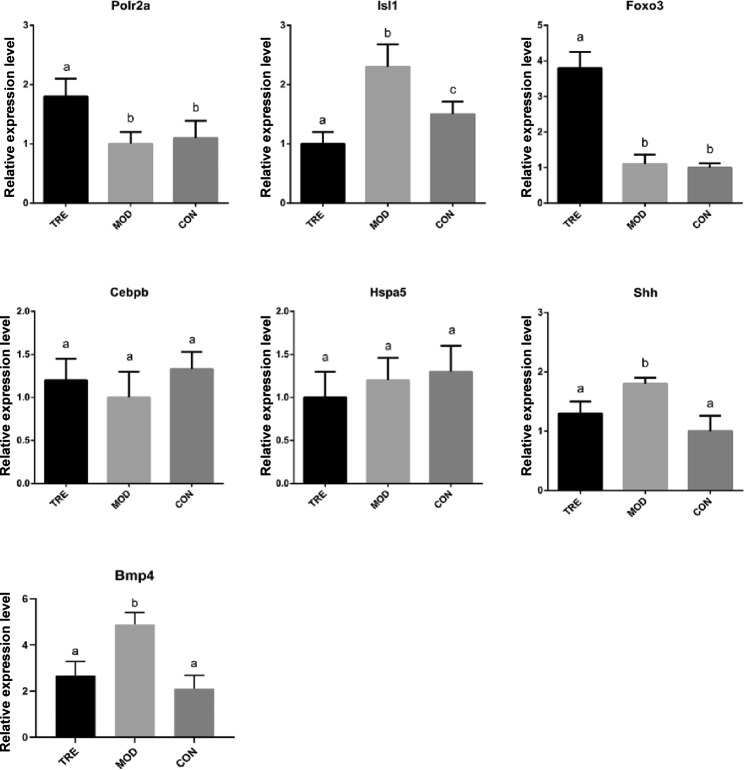
Expression profiles of key DMGs validated using quantitative reverse transcription polymerase chain reaction

Methylation levels of selected key DMGs in the promoter regions were detected (Fig. 7). The levels were evidently similar to the WGBS sequencing results. Additionally, the expression profiles of the selected key DMGs were detected using qRT-PCR (Fig. 8). We found that the expression of *Polr2a* and *FOXO3* was upregulated and that of *Isl1* and *Shh* was downregulated in the TRE group, whereas the opposite result was detected in the MOD group. In the CON group, *FOXO3* and *Shh* expression was downregulated.

## Discussion

### Methylation levels were different between the CON, MOD, and TRE sample groups

As a type of epigenetic modification, DNA methylation regulates gene expression profiles in various biological processes [19, 21]. In our study, we generated and analyzed the genome-wide methylation profiles of CON, MOD, and TRE samples. In addition, protein interactions and associated gene expression were also evaluated for an integrative analysis of epigenetic regulation in IPF. The findings indicated that the methylation levels and methylated regions among the different sample groups were similar, which is consistent with past research [36]. The majority of these C-base methylation patterns were CG, and the top hypermethylated CpGs were likely located within the promoters. DMRs between patients and normal controls have been widely studied; however, DNA methylation and comparisons of Chinese medicine-treated samples have received much less attention. The DMRs were more hypermethylated in the CON vs. MOD group than in the hypomethylation group. In contrast, the DMRs exhibited more hypomethylation in the TRE vs. CON and TRE vs. MOD group comparisons, which sheds light on a potential regulatory mechanism in IPF treatment using TCM.

### DMGs involved in IPF

DMR-associated genes were also detected, and 105 key functional genes were identified. Several IPF treatment-related DMGs have been identified, including *Shc1, Gabra5, Nectin3, Irs2, Ppp1r3d, Shh, Bmp4, Cd14, Epha7, Cdh2, Adcyap1r1, Foxa2, Sema5a, Nup98, Dapk1*, and *Prdm6*. Previous studies have focused on specific genes but not on regulatory networks. In this study, we constructed a PPI regulatory network using the STRING database and filtered the DMGs by their degree values. Seven IPF-related functional DMGs (*Polr2a, Isl1, FOXO3, Cebpb, Hspa5, Shh*, and *BMP4*) were identified for further research. *Polr2a, Isl1, Shh*, and *BMP4* were hypermethylated in the TRE but hypomethylated in the MOD group. *FOXO3, Cebpb*, and *Hspa5* were hypomethylated in the TRE but hypermethylated in the MOD group.

*Isl1*, an LIM homeodomain gene, plays critical roles in segmental patterning as well as cell differentiation, fate determination, and diversity production during embryogenesis [37–39]. The regulatory mechanisms of *Isl1* in tumorigenesis have been extensively studied. It plays an important role in gastric cancer progression and development by regulating the expression of cyclin B1 and B2 (*CCNB1*, *CCNB2*) and *c-MYC* [40]. In addition, *Isl1* functions as a marker for well-differentiated pancreatic neuroendocrine tumors [41], rhabdomyosarcoma [42], and neuroendocrine tumors [43] and also facilitates neuroblastoma development [44]. The role of *Isl1* in the lungs, such as regulating NK2 homeobox 1 (*Nkx2.1*), is necessary for lung lobation and tracheoesophageal separation [45]. The changing methylation levels of *Isl1* in many diseases and developmental processes are concerning. *Isl1* hypermethylation is an independent predictor of disease recurrence and progression in non-muscle invasive bladder cancer [46]. *Isl1* may also help in establishing the epigenetic memory of cardiomyocyte fate commitment [47]. In our study, *Isl1* was hypermethylated in the TRE and hypomethylated in the MOD groups, indicating that methylation levels were related to IPF treatment. The expression profiles of *Isl1* between the TRE and MOD groups were consistent with methylation levels. These results indicate the effectiveness of *Astragalus* and Danshen in IPF treatment. Although the roles and detailed mechanisms of *Isl1* in IPF treatment remain unknown, *Isl1* may be speculated to be a promising therapeutic target.

*FOXO3*, a member of the FOXO transcription factors, mediates many biological processes, such as autophagy in skeletal muscle [48], neural stem cell homeostasis [49], regulation of oxidative stress in erythropoiesis [50], and primordial follicle activation [51]. In addition, an increasing number of studies have indicated that *FOXO3* is a tumor suppressor in many cancers, including gastric [52], colorectal [53], breast [54], and lung cancers [55]. However, the most notable are the roles of *FOXO3* in IPF fibrogenesis. Its expression is downregulated, and it is hyperphosphorylated in IPF fibroblasts. *FOXO3* knockout mice showed heightened susceptibility to the lung-damaging effects of bleomycin, including increased fibrosis, loss of lung function, and higher mortality. In our study, *FOXO3* was hypermethylated, and its expression was downregulated in the MOD but hypomethylated and upregulated in the TRE group. Thus, to some extent, the use of TCM can act on therapeutic targets.

*Shh* signaling regulates many biological processes, such as adrenocortical development [56], the outgrowth and patterning of vertebrate limb buds [57], and medulloblastoma [58]. In addition, both gene targeting and lung-specific transgenic overexpression studies have demonstrated the role of *Shh* during lung development. Deletion of *Shh* in the lung causes failure in branching and growth of the distal lung [59–61]. *Shh* overexpression causes epithelial and mesenchymal cell proliferation to increase, leading to an abundance of lung mesenchyme but no functioning alveoli [59]. According to our results, *Shh* was hypermethylated and its expression was downregulated in the TRE group but upregulated and hypomethylated in the MOD group, indicating its potential regulatory role in IPF treatment. Due to the complex composition of TCM compounds, the in-depth research on the active ingredients of compound drugs and the pathological mechanism of diseases is limited. Currently, more attention is focused on single TCMs and their effective monomer components. However, TCM has synergistic effects on compatibility. Based on the research on TCM pairs, we can carry out the research on the mechanism of multi-target, multi-component, and multi-channel synergistic effects of TCM compounds. In addition, the current research on the treatment of pulmonary fibrosis with TCM mostly focuses on the prevention and treatment of target organ damage from the perspective of intervention pathway, and less on the molecular mechanism and target of TCM on the genetic level. This study deeply explores the mechanism and target of Yiqi Huoxue herbs in the intervention of pulmonary fibrosis through genome-wide methylation. It provides evidence of Chinese medicine intervention in pulmonary fibrosis on the genetic level. However, given the inherent differences between humans and other animals, the data obtained based on animal experiments also have certain limitations, and accordingly require verification in human studies.

## Conclusions

In this study, we investigated whole-genome methylation patterns in the lung tissues of the CON, MOD, and TRE groups. Using this approach, DMGs in different comparison groups were identified, and 105 key functional DMGs were used for further analysis. Based on the methylation levels and gene expression profiles between the TRE and MOD groups, it can be speculated that *Astragalus* and Danshen act on *Isl1, FOXO3*, and *Shh* via regulation at the transcriptional and epigenetic levels during IPF treatment. Our study not only indicates the effectiveness of *Astragalus* and Danshen in treating IPF but also provides various promising therapeutic targets that warrant further study.

### Electronic supplementary material

Below is the link to the electronic supplementary material.

Supplementary Material 1

## Data Availability

The dataset supporting the conclusions of this article is available in the National Center for Biotechnology Information (NCBI) database at the Uniform Resource Locator (URL) link: https://dataview.ncbi.nlm.nih.gov/object/PRJNA934184?reviewer=cfulc06qa2q0dfvida2vsd98m.

## Abbreviations

CON: Control group
DMG: Differential methylation gene
DMR: Differentially methylated region
GO: Gene Ontology
IPF: Idiopathic pulmonary fibrosis
KEGG: Kyoto Encyclopedia of Genes and Genomes
MOD: Pulmonary fibrosis model group
PPI: Protein–protein interaction
qRT-PCR: Quantitative reverse transcription polymerase chain reaction
TCM: Traditional Chinese medicine
TRE: Chinese medicine intervention group
WGBS: Whole-genome bisulfite sequencing
