# Supplementary material for: Genome-wide DNA methylation analysis of Astragalus and Danshen on the intervention of myofibroblast activation in idiopathic pulmonary fibrosis
Source: BMC Pulm Med. 2023 Sep 4;23:325. doi: 10.1186/s12890-023-02601-6 (PMC10478235; doi:10.1186/s12890-023-02601-6)
Supplement: Supplementary file 1 — Supplementary Material 1 [file 12890_2023_2601_MOESM1_ESM.docx]

**Table S1**. List of primer sequences used in the study, analyzed using qRT-PCR at 60 °C.

| Primers | Sequence (5′–3′) |
| --- | --- |
| Tnfrsf17-Fw  Tnfrsf17-Rv  IL-11-Fw | GTGACTATGGCAAGGGTATG  CTTCACATCCCACCAATTAGA  GCTCACCTGTGGCTTATTT |
| IL-11-Rv | GTGAGGAAGACACTGTGAATAG |
| Rasd1-Fw | GCTCACCTGTGGCTTATTT |
| Rasd1-Rv | GTGAGGAAGACACTGTGAATAG |
| IL-1a-Fw | GGCCATAGCCCATGATTT |
| IL-1a-Rv | CCTGCTTGACGATCCTTATC |
| Lair1-Fw | GCCACCAAATGCTTCTCT |
| Lair1-Rv | GTCTTTCCAAGGGCTGATAC |
| miR-676 | CCGTCCTGAGCTTGTCGAGCT |
| miR-2424 | ACAGATCTTTGGTAATCTGATGGCT |
| miR-1247-5p | ACCCGTCCCGTTCGTCCCCGGA |
| miR-3590-3p | TAGCACAATGTGAAAAGAGCTCT |
| miR-9995-3p | ATCTCGGTGGAACCTCCA |
| MSTRG.199-Fw | TGGTGCTTGTGCCTTTAC |
| MSTRG.199-Rv | CTCACCCAACGGAAGGA |
| MSTRG.11560-Fw  MSTRG.11560-Rv  MSTRG.11559-Fw  MSTRG.11559-Rv  MSTRG.30244-Fw  MSTRG.30244-Rv  MSTRG.15160-Fw  MSTRG.15160-Rv | CTCTCGTACTGAGCAGGATTA  GAACGTGAGCTGGGTTTAG  CTCCAGAGGTCCTGAGTTT  CTTCAGACACACCAGAAGAAG  TTAGAGGCGTTCAGTCATAATC  CCGCAGGTTCAGACATTT  GTGCCGCTTTACCCATATT  CCTCAACACCTGTGAGTTTC |

qRT-PCR, quantitative reverse transcription polymerase chain reaction
